# Towards public health ethics

**DOI:** 10.1186/s40985-015-0005-0

**Published:** 2015-05-29

**Authors:** Miguel Ángel Royo-Bordonada, Begoña Román-Maestre

**Affiliations:** National School of Public Health, Madrid, Spain

**Keywords:** Public health, Curriculum, Europe, Ethics, Values

## Abstract

Health is a value, both objective and subjective, yet it is not the only value that contributes to the well-being of persons. In public health, there are different connotations of the term “public” relevant from an ethical perspective: population, government action, and collective action of the community. Ethics seeks to provide a basis for and justify moral decisions and actions. Ethics asks, why should I do it?, and the reply consists of an argument. The type of ethics that underpins applied ethics in general, and bioethics in particular, is civic ethics, a philosophical reflection on the criteria that enable the peaceful coexistence of citizens with different morals. Progress means emancipation as well as an increase of autonomy. However, more is not always better, and now we know that no health intervention, including a public health intervention, is risk-free. The false belief that undergoing a prevention intervention is always better than doing nothing explains, at least in part, that in contrast to bioethics, only recently have the ethical implications in public health practice been given the attention they deserve. Positive externalities in third parties, such as in vaccination programmes or policies to prevent harm to passive smokers, can occasionally justify the potential risks of a public health intervention. It is in such situations where a conflict might arise between the goal of improving the health of the population and the respect for the rights and freedoms of the individual that characterizes the dilemmas in public health ethics. In conclusion, it is necessary to have a public health ethics framework and a professional code of ethics applied to public health. The training of public health professionals in ethics is essential to ensure that they feel more confident when it comes to addressing the sheer range of ethical conflicts that they frequently face in the performance of their duties.

## Introduction

As defined by the World Health Organisation (WHO), health is a state of complete physical, mental and social well-being and not merely the absence of disease or infirmity [1]. This commonly accepted definition has remained unaltered since its publication in 1948, and might have induced some people to equate health with well-being. Indeed, despite having incorporated the concept of well-being into the definition of health, until very recently WHO had neither established what it understood by well-being nor defined indicators to measure this concept. This situation has undergone a radical change with the launch of Health 2020, the European policy framework for health and well-being [2].

Within the framework of Health 2020, the WHO Regional Office for Europe has launched the initiative entitled, “Measurement of and target setting for well-being”, with the aim of soundly defining well-being, analysing the nature of its relationship with health, and defining indicators for measuring it [3]. Most participants of the working group set up to implement the initiative agreed that, “Well-being is multidimensional, includes objective and subjective elements, and there are interactions between health and well-being. Well-being can be seen as a concept and entity in itself (with health as both a determinant and an outcome), and as a composite of various elements (with health as one of them)” [3]. Hence, the relationship between health and well-being can be viewed as bidirectional, in that health influences well-being and well-being influences health. More recently, the WHO Resolution on Indicators for Health 2020 targets has urged the regional director to initiate further work targeted at exploring the means of measuring and setting targets for health and well-being, fully involving Member States; and to continue with the work of the expert group on indicators for Health 2020, with the aim of finalising the development of objective well-being indicators, taking into account social determinants of health and health equity [4].

Accordingly, health should not be equated with well-being, even though health constitutes one of the basic components of well-being and can, in turn, be regarded as a determinant thereof, something that might influence individuals’ self-perception of their personal autonomy and competence. Viewed from this perspective, health is *a* value, both objective and subjective, yet it is not the *only* value that contributes to the well-being of persons.

Bearing this conception of health and well-being in mind, and starting from the different connotations of the term “public”, and the distinction between the concepts of ethics and morals, this paper attempts to explain the *raison d’être* of ethics and justify the need for public health ethics, taking the advances made in recent decades in the field of bioethics as reference.

### What do we understand by public health?

In contrast to the definition of health, which is static, the definition of public health is dynamic. According to Beaglehole and Bonita, public health is one of the collective efforts organised by society to prevent premature death, disease, injury and disability, and to promote the health of populations [5]. A similar definition, drawn up by Acheson, was adopted by WHO and states that public health is the art and science of preventing disease, prolonging life and promoting health through the organised efforts of society [6]. The U.S. Institute of Medicine notes that public health is something that not only concerns public agencies, but also concerns private organisations and communities of individuals, and lays emphasis on assuring a healthy environment, namely, public health is what we, as a society, do collectively to assure the conditions in which people can be healthy [7]. The UK Faculty of Public Health has gone one step further, by incorporating the concept of well-being into the definition, specifying that public health is the science and art of promoting and protecting health and well-being, preventing ill-health and prolonging life through the organised efforts of society [8].

Public health is of a different nature to clinical practice. Whereas the role of the physician centres on diagnosing and treating the illness of an individual, the public health professional must instead analyse the health needs of communities of individuals and the conditions of their social, economic, and physical environment, so as to introduce population-based health promotion, health protection and disease prevention policies. The essential distinctive features of public health are:

- Its interventions are targeted, not at specific persons (who may take advantage of these at an individual level), but rather at groups, such as communities and populations (population education, vaccination, and screening campaigns).
- Its focus is squarely on preserving health, through health promotion, health protection, and disease prevention actions and their consequences, as opposed to the emphasis in clinical practice on restoring health (treatment and cure).
- Its actions are targeted, not only at groups of persons but also at the environment and social circumstances which surround these and influence their state of health.
- It recognises the key role of the State, the need for action of a governmental nature, since many of the actions to preserve the health of the population call for political measures (e.g., regulations, taxation, and mass campaigns).
- It implies collective responsibility for population health, in which all who can contribute are duty-bound to become involved.

In this context, the term “public” has different connotations that must be borne in mind when embarking on an ethical analysis of public health interventions: [9]

- Groups of persons: population,
- Government action: policy and enforcement,
- Collective action of the organised community: social.

Public health invariably implies action, frequently implemented by governments, targeted at the preservation of the health of the population. From this standpoint, public health is in essence paternalistic because it tends to use the power of the State to intervene on behalf of the health of individuals (even where this has not been requested), and utilitarian because it seeks to preserve the health status (something that contributes to the well-being of persons) of the maximum number of individuals possible, ideally the entire population [10]. However, one can never rule out the possibility that actions targeted at promoting health may negatively affect other components of persons’ well-being, a fact of enormous relevance from the point of view of ethics. Lastly, all the above definitions stress the fact that efforts directed at achieving public health goals must be collective, which implies actions based on the participation and cooperation of all the persons and organisations, governmental or non-governmental, public or private, involved in public health, whether or not affected by such actions. From this perspective, public health also has a social and communitarian essence [10]. In brief, the above-mentioned three dimensions or connotations of the concept “public” (population, political, and social) correspond to public health’s different facets, i.e., utilitarian, paternalistic, and communitarian.

### What do we understand by ethics?

Ethics is moral philosophy, a critical-rational reflection on morals. Ethics, more than being descriptive of a given society’s moral codes, has a normative pretense in that it asks whether morals are legitimate, whether they have some underlying justification; in other words, beyond establishing whether rules and values are in force, ethics reflects on their validity. Ethics thus mulls over morality in general to ascertain the conditions of its validity and helps render the different historical morals respectable. Morals are cultural products, relative to historical contexts. Ethics cannot deny the reality of moral pluralism: it holds that many moral options are valid but that not all are valid or even equally valid.

The moral question (from the Latin, *mos-moris*, habits, customs) is, *what* should I do?, and the reply consists of proposing an action (or an omission). Ethics (from the Greek, *ethos*, character) asks, *why* should I do it?: it positions itself at a secondary, contemplative level and the reply consists of an argument. Hence, Aranguren was right when he summarised the question of the distinction between ethics and morals by saying that morals are lived and ethics are thought [11].

What then does ethics contribute? Since it aspires to universality, in its guise as normative ethics it seeks to provide a basis for and justify moral decisions and actions, by going beyond the mere first person singular, the “I”. Ethics maintains that every moral good is the object of desire of an autonomous will, inasmuch as if it is something that someone desires for himself, because he consider it a valuable good, he must at the same time desire it for everyone everyone else. Thus, the criteria of universalisation and of personal autonomy and commitment are key criteria of normative ethics, which seeks to guide moral actions by being constantly prepared to scrutinise them.

Anyone who critically examines his own morals must distance himself from them; Socratically, he must doubt them, questioning whether such customs are the best, something that entails an awareness that reflects and overcomes unconscious attachment to morals received through acculturation. Accordingly, ethics, in the form of critical-rational reflection, requires a developed moral conscience of a postconventional level on the Kohlberg scale (Lawrence Kohlberg’s stages of moral development constitute an adaptation of one of Jean Piaget’s psychological theories. The theory holds that moral reasoning, the basis for ethical behaviour, has six identifiable developmental stages, each more adequate at responding to moral dilemmas than its predecessor. Kohlberg’s scale is about how people justify behaviours and the general hypothesis is that moral behaviour is more responsible, consistent and predictable from people at higher levels) [12].

On this postconventional level, one develops a critical capacity vis-à-vis the s*tatus quo*, the “establishment”. At this level, one is capable of taking up a stance against conventions and fears neither autonomy nor being rejected by the group. Conventions are criticised for the inadequacy of the criterion that equates the socially current with the ethically valid. The preconventional is also criticised, with the warning that just because something is liked, does not mean that it is correct. Thus, only on this postconventional level is one able to assume the criterion of universalisability, where the key question is whether one can desire something both for oneself and for everybody else.

The ethics that is needed in technical societies -dizzyingly changing societies with impacts worldwide- is normative ethics, and not meta-ethics, which is, in turn, a reflection on ethics, on its manner of proceeding and reasoning. Since complexity is the hallmark of societies such as ours, in which interdisciplinarity—an essential characteristic of public health—becomes inevitable for being able to better manage risks, applied ethics arises in order to delimit reflection and render it fruitful in a specific sphere of activity. A knowledge society, such as ours, is thus in more need of ethics than it is of mere morals. Consequently, it is a moral duty to be efficient and intelligent –to exercise and develop the abilities and skills with which we are endowed [13].

The type of ethics that underpins applied ethics in general, and bioethics in particular, a type that is required to be of a lay nature, suited to managing the conflicts of coexistence in morally plural societies, is civic ethics, a philosophical reflection on the criteria that enable the peaceful coexistence of citizens with different morals [14]. Civic ethics is *minimal* ethics: it does not lay down *all* the norms to be followed but only (an “only” that denotes modesty and not a lack of importance) such norms as can be demanded of all citizens to create coexistence, one that is fitting for a society that respects human beings, beings with rights (indispensable elements, primary and priority, for being able to live humanely) and, by extension, with duties (obligations and commitments toward both human and other non-human beings).

We need this type of ethics more than ever for the public space that will allow us to exist and coexist peacefully, tolerating perspectives that we do not share [15]. From this public ethics viewpoint (of citizens and the institutions that serve them), the issues of quality of life and preferences (which vary from individual to individual, and over the course of any given individual’s lifetime) and of justice and dignity (of any person at any place and time) arise. Popper reminded us that the open, free, and tolerant society has its enemies, in the form of fundamentalism and fanaticism, which insist on imposing a certain way of looking at the world and assessing life [16]. Yet a further risk is that liberalism defends autonomy to the point of converting it into personal preferentism, which ends only where the preferences of others begin, thereby leading to both the normative moment and that of universalisation being missed. Hence, one person limits himself to expressing his preference and the others to respecting it purely because it is his.

This is truer today than when Francis Bacon stated in the 16^th^ century that knowledge is power, since great changes are born of knowledge. Part of the problems of the 20^th^ and 21^st^ centuries are not, as is so often bemoaned, entirely due to a lack of ethical and political will: they are also due to a lack of knowledge and the organisation of such knowledge [17]. Knowledge societies, which are both thinking societies and societies shaped by thought, make this effort to reflect on their own practices: on the one hand, because the frenzied speed of society makes it necessary to keep abreast of the times and to foresee, and not merely reactively resolve the problem outlined above; and on the other hand, because, as Jonas points out, the technology and power of intervention today are far riskier than those of earlier societies and technologies. This, taken together with the irreversibility of some consequences, is sufficient cause for caution, in order to avoid acting rashly in the name of acritical techno-scientific progress [18].

Technology opens up a broad scope of intervention: on being masters of our own destiny, thanks to such technology, we assume a power for which we have to be accountable. Technocratic euphoria still prevails, that is, what is scientific is good *per se*, inasmuch as it is axiologically neutral and disinterested; and everything new is better, from a concept of progress that is philosophically -insofar as it goes unquestioned- clumsy. From an ethical point of view, progress means emancipation -ridding oneself of superstitions, ignorance or physical and social impediments- as well as an increase in degrees of autonomy (of opportunities and capacities). Now we know, however, that more is not always better, and hence iatrogenesis, brought about by medicine itself.

Furthermore, the contributions of philosophy explain the need for public ethics and the advent of applied ethics. Specifically, contemporary philosophy is characterised by four aspects: the pragmatic, the hermeneutic, the linguistic, and the applied. In effect, this amounts to thinking pragmatically to solve problems, problems that are addressed from the standpoint of the experience and interpretation of a subject situated in a cultural, historical, material and grammatical context. Accordingly, this amounts to a criticism of philosophy for becoming embroiled in abstract discussions about themes and meta-themes, and an *assiduous* shift to *real* problems, to the concrete, to the world of the living. Toulmin adjudged the situation rightly when he said that bioethics saved the life of moral philosophy, which had been left to sterile discussions, by spotlighting it and placing it at the centre of life and general interest [19].

Together with these circumstances, mention should also be made of the following factors that explain the rise of applied ethics in general and of bioethics in particular, back in the 1970s, and the need for their institutionalisation in different committees.

Firstly, there is the corporate or organisational environment. There are hardly any professionals left who can really practise their professions as self-employed university-qualified practitioners, which is the model that many have in mind when entering a professional society or association. The necessary investment in training, interdisciplinary teams, and technical resources acts as a major hindrance to such autonomy. Twentieth century society is one of teams, organisations, and networks. All this renders it necessary to think of norms and, over and above those that are purely deontological and legal, of ethical ones: beyond statutory regulation, there is place for self-regulation.

Yet serving someone as a salaried employee, and serving another, solely by virtue of having a qualification, but who, in H.T. Engelhardt’s famous phrase, is a moral stranger in whom one has no trust, renders the relationship among the agents involved in bioethics (e.g., patients, professionals, professional societies and associations, health organisations, insurance companies, and parliaments far more complex) [20]. Conflicts of interests thus become inevitable.

Secondly, the obsolescence of approaches, on the one hand, and the new skills and techniques, on the other, tend to entail a certain dehumanisation in some professions, which though very focused and concentrated on the technical side, nevertheless gradually lose sight of the essential, the mission, the task with which society has entrusted them. So much so indeed that some say, *many medical actions are performed* but *this is not being* a doctor: the virtues, which are attributes of the character, are forgotten.

Service quality demands more than just satisfying the customer, since the latter is no longer always right, because we are talking of specialised services with social, ecological and economic impacts. If we do not wish to participate in excessive acts of faith (in the ingenuous goodness of the expert or in the much vaunted social responsibility of organisations), we are obliged, as professionals and organisations, to reason our decisions and to be deserving of the trust placed in the professional, the profession, the sector and public policies.

For all of the above reasons, ethics is essential.

### What do we understand by bioethics?

Bioethics conceives health science, and the different specific disciplines that intervene in it, such as medicine, nursing, dentistry, physiotherapy, and others as regulated activities. As a result, the respective purposes, missions, views, values, contexts, impacts, interests and powers must be established and specified. Bioethics thus makes explicit the inevitable interests that impregnate every human action. Bioethics has overcome the prejudice of a positivism (technical issues are free of values), which is still deeply rooted in many disciplines due to a lack of introspection into their own practices [21].

A number of codes and declarations have been crucial for the development of bioethics, including the Nuremberg Code (1947), the Belmont Report (1978) and the Helsinki Declaration of the World Medical Association (1964), with successive revisions (the latest in October 2013); in these, reference is made to the ethical principles that should govern research on human beings, such as respect for persons, beneficence and justice. These were followed by: the Oviedo Convention (1997) for the Protection of Human Rights and Dignity of the Human Being with regard to the Application of Biology and Medicine; and the UNESCO Declaration of Universal Human Rights (1997). In addition, the UNESCO also issued the Universal Declaration on Bioethics and Human Rights in 2005. All of these documents form part of the body of guidance that highlight the need to define and delimit what should and should not be done (even though it might be technically feasible).

Bioethics, as a specific body of knowledge, came into being in the United States at the beginning of the 1970s [22]. Three key names are to be found at its origin: André Hellegers, founder of the Kennedy Institute of Bioethics; Daniel Callahan, founder of the Hastings Centre; and Van Rensselaer Potter, who coined the name “Bioethics” in the article, “Bioethics: the science of survival” (1970), and the book, “Bioethics: bridge to the future” (1971). In Europe, bioethics made its institutional *début* in the mid-1980s. Names, such as Jean-François Malherbe (creator of the Centre d’Etudes Bioéthiques in Brussels), Nicole Léry (Lyon Centre), Patrick Verspieren (Paris Centre), Francesc Abel (Borja Institute) and Diego Gracia (Complutense University of Madrid) feature among its first exponents.

Despite the fact that bioethics arose with a generic approach, its subsequent development gradually became more closely aligned with medical ethics, focusing on the doctor-patient relationship and the sphere of biomedical research. Nevertheless, the continuous expansion of its horizon, with social, political and legal implications, among others, means that in general there is a growing trend to speak of ethics as such [23]. Even the first institute with the name of bioethics, the Kennedy Institute, changed its name and is today called “The Kennedy Institute of Ethics”.

In brief, bioethics arose closely linked to the following context:

- morally plural societies, where one has to debate about what is good/bad, the correct/incorrect, and where legality is what it is but could be otherwise;
- democratic settings, characterised by the rule of law, where tolerance is a duty;
- organisations that require qualified professionals who, rather than working as self-employed university-qualified practitioners, are salaried employees; and,
- knowledge societies in which uncertainty and complexity require that the public decisions that must be made are taken in public discussion forums.

In bioethics, the problems, those affected in their contexts, and their interests are the transcendental triad when it comes to deciding on the action to be taken. In clinical ethics committees, problems are deliberated and discussed in an institutionalised interdisciplinary debate, with the health professionals who have the specific technical knowledge of their medical specialisation, with legal advisers, with social workers, with patients’ representatives, and with experts in ethics. This is done by weighing the principles (fundamental values), appraising the actions at hand in proportion to the available resources (if it should be done, it can be done), avoiding the consequences that are undesirable, and fostering those that are desirable, which are so by virtue of their being in accordance with the values whereby we, as a society, wish to be characterised [23].

Bioethics is institutionalised in *dialogue forums of discussion and debate, plural, and independent* of the directives of the organisations or governments that create them. Hence, bioethics committees were set up to recommend the best courses of action to professionals, citizens, and parliaments in the light of currently available knowledge. What bioethics seeks is to manage the tension between risks and benefits, and although it is confined to making recommendations, it also recommends that some of these be made into laws. Bioethics has shown that it is possible for conflicts, values, and interests to be seriously discussed without automatically succumbing to factual consensus or inefficiency. In bioethics there is thus a desire for normativity, efficiency, and legitimacy.

Methodical doubt, inherent in the very business of philosophy, also forms part of bioethics. But a child of existentialism, the latter commits itself in discussion fora where, regardless of the ideology of the dominant political party, decisions are recommended and reasons are given in an attempt to mediate in the quandary that the uncertainty and complexity of the problems generated. While solutions are sometimes be grounded in evidence-based medicine, others might also be grounded in prudence resulting from science-based ignorance: all of which makes transdisciplinarity and humility essential.

There are occasions when no other course is open to this type of ethics in these technological times than Socrates’ old realisation that, “I only know that I know nothing”. Even so, it commits itself to a dialogic search for the best intervention to be made, given the settings, the values of those involved, those actions that are feasible, and the foreseeable consequences, after a complicated appraisal based on these values. We were therefore in need of ethics of finitude, of caution, which took on the obsolescence of approaches, the inescapable historical perspective. And it so happens that it serves to manage risks and meet the challenges of the wilderness into which we were cast by the contemporary age, which left us without the certainties of strong categories.

Of the various bioethics schools, it was principlism that enjoyed the greatest success [22]. Agreement on the principles, as set forth by Beauchamp and Childress [24], has meant that conflictive cases could be illustrated and efficient ethical-legal recommendations proposed in the course of medical ethics and biomedical research.

Principles, as the term implies, are the assertions that must necessarily be assumed on embarking upon any course of reasoning of an ethical nature, provided that such principles are not deduced from something prior, something that precedes them, because then they would not be principles *per se*: what one does by expounding them is to highlight the basic values, the inescapable conditions in any bioethical line of reasoning.

Beauchamp and Childress define the *principle of beneficence* as, “the moral obligation to act for the benefit of others” [24]. They contend that there are obligatory principles or rules of beneficence, namely: “1) to protect and defend the rights of others; 2) to prohibit causing harm to other persons; 3) to remove conditions that may cause harm to others; 4) to help persons with disabilities; and 5) to rescue persons in danger”. Furthermore, they remind us that the physician is under an obligation *to do no harm* (physically, mentally and/or morally) to his patients. From this principle the following set of rules is also drawn: “1) do not kill; 2) do not cause pain or suffering to others; 3) do not incapacitate others; 4) do not cause offence; and 5) do not deprive others of the goods of life.” Both principles, namely, non-maleficence and beneficence, must be evaluated from the standpoint of the physician, who is familiar with the disease and its treatments, and of the patient, since it is from the perspective of his personal life plan, and, thus, respect for his autonomy, the other major bioethical principle, that one determines whether a given action will be beneficial or prejudicial. Justice, the principle that alludes to the distribution of resources, is the reference point when it comes to weighing the principles, and the decision will vary according to whether or not there is a public health system in place.

This approach, while undoubtedly necessary, will not suffice: there is still a need for professional virtues, construed as the way in which such professionals act, insofar as they involve themselves in the quality of the service (which requires a type of training) and the analysis of each particular case.

To recapitulate, it can be concluded that bioethics, above and beyond ideologies and cultures, is based on:

- a worldwide body of civil ethics whereby human rights are recognised as shared moral convictions, morally respected, and with legal guarantees under an effective and just international law;
- the ethical responsibility of all professionals involved in health care and research;
- the joint ethical responsibility of organisations and citizens; and,
- a review of health policies and procedures when it comes to taking decisions.

We feel that all the above advances in bioethics could provide an excellent starting point for addressing the issue of public health ethics.

### Some ethical concerns in public health

In contrast to the substantial body of bioethics, only recently have the ethical implications in public health practice been given the attention they deserve [25, 26]. The reasons cited to explain what amounts to such a late development are tied to the origins of preventive medicine, associated with health policing tasks and the false belief –widespread among the population- that undergoing a prevention intervention is always better than doing nothing, assuming that preventive activity is risk-free or that any undesirable consequence flowing therefrom is more than offset by the benefit obtained [27]. Yet no health intervention, including a preventive or health promotion intervention, is risk-free. Although the harm caused to participants by public health interventions might be minimal, the impact can be extremely relevant, since such interventions tend to be targeted at a very large number of persons, most of whom are healthy. The presence of positive externalities in third parties can occasionally justify the possible harm caused by an intervention [25] such as the herd effect of vaccination programmes, which acts as a protection against the disease in persons who have not had the possibility of being vaccinated due to medical or immunological contraindications, access barriers or any other reason [28], or the damage preventable to the health of passive smokers by a ban on smoking in public places [29]. It is in such situations where a conflict may arise between the goal of improving the health of the population and the respect for the rights and freedoms of the individual that characterises the dilemmas in public health ethics [30].

In contrast, in preventive interventions where there are no positive externalities, it is far more difficult to justify an intervention targeting apparently healthy persons, so that, if anything, informed consent becomes even more relevant than in routine clinical practice. For instance, breast cancer mammography screening programmes involve adverse effects associated, either with the technique, such as discomfort and radiation, or with false positives, such as unnecessary tests, anxiety, overdiagnosis, and overtreatment [31]. Often, programmes tend to inflate the information about the benefits, and restrict or minimise that relating to the harm, making excessive use of persuasive language which limits the autonomous decision-making capacity of the interested parties vis-à-vis mammography screening [32]. This is particularly worrying if one bears in mind that social, cultural, and identity values can alter the perception of risk among women, by overestimating the threat of breast cancer in comparison with other much more lethal conditions, such as cardiovascular diseases [33]. Although some progress has been made to improve the information furnished to candidates for screening, such as the evidence-based leaflet for lay people published by the Nordic Cochrane Centre [34], there is still a long way to go in what is a fertile field for debate between experts in ethics, public health, and communications. A good example of this are the reactions to the publication in 2009 of the U.S. Preventive Services Task Force recommendation against routine screening mammography in women aged 40 to 49 years [35]. Despite the strength of the scientific evidence that underpinned the recommendations, many politicians and physicians questioned them, and a survey found that 84 % of women aged 35 to 49 years considered ignoring them [33]. Daniel B. Kopans, a radiology professor at Harvard Medical School, stated the following in the *Washington Post*, “Tens of thousands of lives are being saved by mammography screening, and these idiots want to do away with it. It’s crazy -- unethical, really.” [36] The position of the American College of Radiology was to recommend annual mammography screening starting at age 40 years [37]. Apart from the strength of the scientific evidence that underlies these pronouncements, it seems questionable to use bombastic phrases to proclaim the alleged benefits of an intervention, overlooking the risks and any reference to individual decision, especially when those making these types of claims are subject to the conflict of interest stemming from the economic benefits that they obtain on implementing the intervention. Similarly, the Swiss Medical Board’s recommendation, made public on 2 February 2014, against the introduction of new systematic mammography screening programmes (www.medical-board.ch), was rejected by a number of Swiss cancer experts and organisations, some of which called the conclusions “unethical”. In reply to this criticism, Nicola Biller and Peter Jüni said: “From an ethical perspective, a public health programme that does not clearly produce more benefits than harm is hard to justify. Providing clear, unbiased information, promoting appropriate care, and preventing overdiagnosis and overtreatment would be a better choice” [38]. Although it seems a reasonable conclusion, the article did not include an explicit discussion of the ethical arguments underpinning this statement. No matter who invokes it, ethics should never be used as a weapon to bring down an opponent but rather as a way of solving the conflicts one has to confront in daily practice.

In health promotion, where the goal is to foster healthy habits, the risks of interventions are not quite so evident but they can nevertheless exist. For instance, obesity prevention campaigns could stigmatise the persons affected if they represent these as being exclusively responsible for dietary habits which are in great measure determined by social, economic, genetic and cultural factors, outside the control of the individual, the so-called “victim blaming” [39]. In an ideal world, with competent persons free of negative influences, and in the absence of externalities, it would be good enough to provide valid, relevant, and comprehensible information about the relationship between lifestyle and health, present and future, and let each individual freely decide how to act, in accordance with his preferences. Yet reality is very far from being like this. Frequently, individual decisions are not altogether voluntary, either because the persons concerned are influenced by the environment in which they live, or because they are unable to foresee the consequences of their actions, are not well informed, or are subjected to negative external influences at odds with their interests [40].

Some health promotion interventions, such as the compulsory wearing of a crash helmet when riding a motorcycle, are justified on the basis of negative externalities for the society arising from the economic costs of the treatment and rehabilitation of accident victims [41]. Even if drivers were willing to take out a private insurance policy that covered these costs, the suffering caused to the relatives and friends of accident victims could be cited to justify the measure. Regardless of whether or not these types of externalities exist, health promotion interventions often give rise to objections for attacking individual freedoms, interfering in personal decisions (negative concept of freedom, such as absence of interference) and the free market [42]. Such critics do not, however, seem to be concerned by the interference in personal decisions caused by changes to the environment and the mass use of manipulative advertising by industries which manufacture products that are harmful to health [43]. Under a positive concept of freedom, construed as the ability to act in one way or another, and in the absence of externalities, health promotion interventions could be justified by reference to their effectiveness in enhancing individuals’ real capacity to choose freely (ably, well-informed, and free of controlling influences), by developing their personal capacities through community-based interventions with empowerment techniques [44], counteracting corporate-interest-led manipulation of information through social protest and marketing campaigns, or changing the environment to render healthy options more accessible [45]. In this context, public health interventions take the form of restricting the advertising of energy-dense, nutrient-poor foods aimed at children [46], developing the nutrition traffic light [47], and limiting the use of hydrogenated fatty acids in the manufacture of processed foods in Denmark [48]. Lastly, transparency and citizen participation in the process of drawing up public health policies, through democratic channels, are essential for legitimising interventions aimed at modifying the behaviour of individuals.

As discussed above, the proposed interventions sometimes clash with the interests of certain corporations (tobacco, alcohol, and food industries), which lobby politicians and public health officials in an attempt to get these to redirect policies towards the defence of their private interests, in detriment to the health of the population, the so-called “corporate capture”. Two examples of this include the UK’s reversal on setting a minimum unit price for alcohol [49] and the failure of the new Spanish co-regulation code for food advertising to protect children under 15 years of age [50]. When the pressure of private corporations is felt, public health officials, usually civil servants, are confronted by a conflict between their duties, on the one hand to the governing class who decide the policies and, on the other to the citizens whom they are bound to serve in accordance with the dictates of their best professional judgment [51]. It is not easy to resolve this conflict in a context where the rule is to work with a lesser or greater degree of scientific uncertainty. Some authors have sensibly proposed that public health officials are responsible for trying to persuade the health authorities (their superiors) about alternative courses of action that are more in accordance with public health values and the best scientific and professional judgment [52]. However, once the decision has been taken by the institutions democratically entitled to do so, public health officials are under an obligation to implement the intervention in question, provided that it is lawful and ethically acceptable, refraining from questioning it publicly, since this would amount to sending out a contradictory message which would undermine the population’s trust in public institutions. Yet, this in no way implies that public health officials are obliged to do a job of advocacy, attempting to convince the public about the marvels of a given intervention that does not entirely accord with their professional values and/or judgment. Neither does it mean that public health officials cannot act –regardless of their status as civil servants- as citizens or public health professionals, promoting alternative policies to improve public health based on their professional and scientific knowledge, values and judgment.

## Conclusions and Recommendations

While the use of bioethics in the field of public health could be addressed, it must be said that the doctor-patient relationship in clinical practice is of a different nature to the relationship between the population and the public health officials and professionals who undertake the preventive or health promotion interventions [53]. Firstly, in clinical practice, the recipients of the interventions are patients who seek care, whereas in public health these are, at least apparently, healthy persons who have not sought the intervention offered to them. While all the patients will obtain some benefit, larger or smaller, from the treatment, only a variable, though generally small, percentage of the persons who participate in a public health programme will obtain a benefit in the future, something that has come to be called the prevention paradox [54]. As indicated above, it is logical to think that the latter’s willingness to assume the possible risks of the intervention is not the same as that of sick persons. Secondly, among public health officials, there is a political component in the form of the health authority, with legal capacity in certain instances, to take action targeted at the individual or the environment. This capacity to restrict the autonomy of the individual can, as seen above, come to be justified on the basis of the externalities, positive or negative, induced by the intervention in third parties [25].

*Prima facie* there are four principles of bioethics, though it is usually accepted that a certain degree of primacy must be accorded to the principle of autonomy, which has come to be known as the first among equals [55]. The reason for this resides, firstly, in the fact that the defence of the principle of autonomy lies at the origin of medical ethics and, in a broader sense, of bioethics; and also in the fact that the principle of autonomy is seen as an integral part of the other three principles. It has thus been argued, rightly, that deliberations and ethical recommendations made in the field of bioethics (clinical practice and biomedical research) are not directly applicable to the field of public health, where the principles of beneficence and justice would have pre-eminence, since their mission is to preserve and promote the health of the population [53]. Furthermore, there are additional values and considerations relevant for public health ethics, such as solidarity, transparency, pluralism and community’s perspectives, among others [56–58]. The foundations of bioethics and the endorsement of principlism are important elements for the development of ethics in public health. No doubt, we need to consider other complements.

Traditionally, public health interventions with ethical implications have lacked ethical analysis or had to contend with conflicting and ambiguous ethical principles [59]. Particularly noteworthy is the scant participation of the target population at which public health interventions are directed, when it comes to reviewing public health strategies and drawing up the pertinent interventions [60]. The ethical conflict *par excellence* in public health arises from the tensions between the common good and the rights of individuals [30]. In this context, it is the community view of public health, with its insistence on justifying public health policies and ensuring the participation of those affected by them, that should be required to reduce these tensions, by seeking to make the common good compatible with individual freedom. Beyond a paternalistic approach, the public health of the future should tend to seek the commitment and participation of the public in the construction of a healthy environment, which would ensure equality of opportunity to all for the purpose of achieving an optimal health status [61].

In conclusion, it is necessary to have a public health ethics framework that values things other than the four principles and codes of ethics (a list of conduct statements for public health professionals), and that takes into consideration this discipline’s idiosyncrasies and the specific traits that distinguish it from clinical practice and biomedical research [62, 63]. The training of public health professionals in ethics is essential to ensure that they feel more confident when it comes to addressing the sheer range of ethical conflicts (e.g., public-private partnerships, allocation of scarce resources, collection and use of data, policy-making processes, and relationships with health authorities and other government officials) which they frequently face in the performance of their duties [60].

Miguel Ángel Royo-Bordonada holds a PhD in medicine and specialist in preventive medicine and public health. He is Professor in the Spanish National School of Public Health, located in Madrid. He is chair of the MPH Academic Board at the National School of Public Health, the Spanish Interdisciplinary Committee on Cardiovascular Prevention, and the Aspher Working Group on Ethics and Values in Public Health. His areas of interest are food policy, obesity and cardiovascular prevention, corporate capture and public health ethics.

Begoña Roman-Maestre holds a PhD in philosophy. She is Professor in the Faculty of Philosophy at University of Barcelona. She is member of the consolidated research group by the Generalitat of Catalonia “Aporia: Contemporary Philosophy, ethics and politics.” She is Chair of the Ethics Committee for Social Services in Catalonia and member of the Bioethics Committee of Catalonia. Her area of expertise is applied ethics and bioethics.
